# Effect of aerosolized nicotine on human bronchial epithelial cells is amplified after co-administration with cannabidiol (CBD): a pilot in vitro study

**DOI:** 10.1186/s40360-020-00418-1

**Published:** 2020-06-04

**Authors:** Noel J. Leigh, Maciej L. Goniewicz

**Affiliations:** Department of Health Behavior, Division of Cancer Prevention and Population Sciences, Roswell Park Comprehensive Cancer Center, Buffalo, NY USA

**Keywords:** Electronic cigarettes, E-cigarettes, Electronic nicotine delivery systems (ENDS), Inhalation, Toxicity, Cannabinoids

## Abstract

**Background:**

Population-based studies suggest increasing rates of concurrent use of vaping products that contain either nicotine or cannabinoids. The aim of this pilot study was to test in vitro the acute inhalation toxicity of vaporized flavored and unflavored nicotine solutions co-administered with cannabidiol (CBD).

**Methods:**

Bronchial epithelial cells (H292) were exposed directly to aerosol generated from electronic cigarettes refilled with propylene glycol only, unflavored nicotine solutions in propylene glycol with and without CBD, as well as to solutions containing only CBD. Cells were also exposed to a commercially available flavored solution containing nicotine and CBD. The in vitro toxicological effects were assessed after exposure using the following methods: 1) a trypan blue exclusion assay (cell viability), 2) neutral red uptake assay (metabolic activity) and 3) ELISA (concentrations of inflammatory mediators).

**Results:**

Unflavored solution containing only CBD was significantly more cytotoxic than unflavored solution containing only nicotine. Unflavored solution containing both CBD and nicotine was significantly more cytotoxic than unflavored solutions with only nicotine. Levels of released cytokines were significantly higher when cells were co-exposed to nicotine and CBD as compared to cells exposed to only nicotine or only CBD. Overall, flavored products showed increased toxicity as compared to unflavored solutions.

**Conclusions:**

This pilot in vitro study suggests independent and additive toxic effects of vaporized nicotine and CBD. Observed toxic effects are accentuated by flavorings. Future studies are needed to determine the potential long-term health consequences of concurrent use of vaporized nicotine and cannabis products.

## Background

Electronic cigarettes (e-cigarettes), or electronic nicotine delivery systems (ENDS), were developed as potentially less-harmful nicotine delivery products than combustible tobacco cigarettes. While ENDS have become highly effective in delivering nicotine, population based studies have shown that these products have also been used to vaporize other psychoactive substances, including cannabinoids [1, 2]. Population-based studies have shown that a significant proportion of tobacco smokers also use cannabis [3, 4]. Although cannabis-derived products are becoming de-criminalized throughout individual states in the United States [5], products containing a mixture of cannabinoids are still classified as Schedule 1 substances under the United States Drug Enforcement Agency Controlled Substances Act. However, products that only contain cannabidiol (CBD) are promoted and marketed without restrictions based on a claim that CBD-only products are derived from hemp, and not from cannabis. As marijuana smoking remains the most popular way for delivering cannabinoids to the body, very little research has been performed to examine delivery and health effects of vaporized cannabinoids, including CBD.

Cannabidiol was discovered in the early 1930’s and has been found to have anti-convulsive [6–8], anti-psychotic [9, 10], anti-inflammatory [11, 12] and sedative effects [13, 14] in vitro and in vivo. While these studies showed positive effects of CBD when administered orally, topically or via intraperitoneal injection, few studies to date have examined health effects of CBD when inhaled using ENDS devices.

Potential respiratory effects associated with co-use of nicotine and CBD have not been studied. In this pilot study, we used a physiologically relevant in vitro model to examine respiratory effects of inhaling aerosols containing nicotine with and without CBD, as well as to determine if there are any additive effects associated with combined used of nicotine, CBD with and without flavorings.

## Methods

### Commercially purchased ENDS device and refill solutions

A puff activated eGO tank (SmokeTek), was purchased online for this study. This product had a fixed battery output voltage of 3.8 V and the coil in the CE4 tanks had an average resistance of 4.0 Ω resulting in 3.6 W of power. CBD-containing liquid of a single flavor labeled “Easy Rider” and a labeled CBD concentration of 50 mg/30 ml (1.7 mg/ml) was purchased online. While the flavor classification of this liquid was unknown, we speculate it has a fruity flavor based on the smell and GC/MS profile of detected flavoring chemicals. We also purchased one unflavored CBD liquid labeled “pure” CBD 1000 mg/30 ml (33.3 mg/ml).

### Lab-made and lab-modified refill solutions

**Fig. 1:**
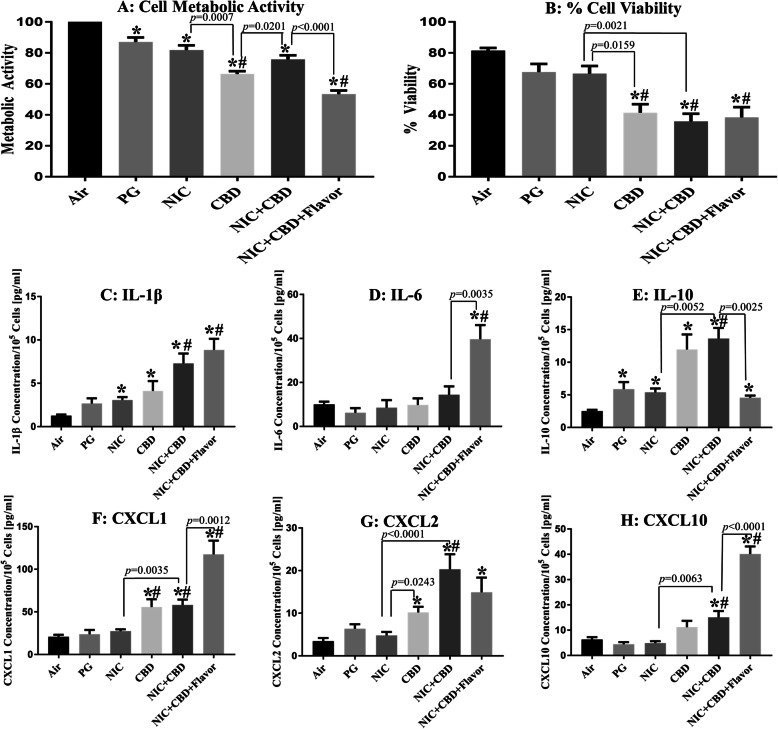
Comparison of cellular toxicity and levels of released inflammatory mediators (cytokines) from H292 bronchial epithelial cells directly exposed at the air-liquid interface to 55 puffs of nicotine and CBD aerosols. All aerosol was generated from an eGO tank system, with battery output voltage set to 3.8 V and refilled with PG-only solution with the same nicotine and CBD concentrations (1.7 mg/mL). * indicates significant difference from the air control and # indicates significant difference from the PG solvent control

Refill solutions containing propylene glycol only (**PG**, solvent control, 99 + % Acros Organics), PG with nicotine only (1.7 mg/ml, **NIC**), PG with CBD only (1.7 mg/ml; **CBD**), PG with nicotine and CBD (1.7 mg/ml each; **NIC + CBD**) as well as flavored liquid (Easy Rider) with PG, nicotine and CBD (1.7 mg/ml each; **NIC + CBD + Flavor**) were tested (Fig. 1). PG with CBD only and PG with NIC + CBD was made using commercial liquid containing a listed 33.3 mg/ml CBD concentration. This product was diluted with PG to create a solution with CBD concentration of 1.7 mg/ml. Nicotine (99 + %, Acros Organics) was added to the commercially purchased flavored CBD liquid to create **NIC + CBD + Flavor** solution with the equal CBD and nicotine concentrations of 1.7 mg/ml. Nicotine was also added to PG to create a 1.7 mg/ml solution (**NIC**).

### GC/MS analysis of refill solutions

Flavoring chemicals were identified in each liquid using a gas chromatography/mass spectrometry (GC/MS) method, as described previously [15]. CBD concentrations were compared with the same peak area of analyzed samples. All commercially purchased CBD liquids were listed as industrial hemp derived and contained no delta-9 tetrahydrocannabinol (THC) as confirmed by GC/MS analysis.

### Generation of ENDS aerosol

Aerosol from the eGO ENDS was generated using a Borgwaldt LX-1 (Richmond, VA) single-port piston-operated smoking machine. The Health Canada Intense (HCI) puffing protocol was utilized with the following conditions: 2 s puff duration, every 30 s, with a 55-mL puff volume. The puffing protocol was used continuously for 55 puffs or 30 min following protocol described previously [15]. Air-exposures (air control) were run during each experiment.

### Cell exposure conditions

The NCI-H292 cell line (ATCC) was used for all experimentation. Cells were exposed directly to freshly generated aerosol in an air liquid interface (ALI) as described previously [15]. During cell exposure to air or ENDS aerosol, fresh media was cycled over the basal side of the permeable support at a flow rate of 5 mL/min. After exposure, 1 ml of culture media was added to the apical side of the cells grown on permeable supports. Then we waited 2.5 h before we examined endpoints, see toxicity assays below. While this system, like any ALI system, does have its limitations we tried to overcome these by exposing cells to an air control and PG control for each experimental day to ensure equal exposure.

### Metabolic activity

Metabolic activity of exposed H292 cells was measured by Neutral Red Uptake Assay [16, 17] as described previously [15]. Briefly, the top and bottom surface of cells was covered with a diluted neutral red dye. After 2.5 h each permeable support was washed with PBS, then a de-stain solution was added to top and bottom of the permeable support then rocked for 10 min. This de-stain solution was added to a 96-well plate and measured with a BioTek Epoch spectrophotometer at 540 nm in triplicate.

### Cell viability

Cell viability was measured by Trypan Blue Assay as described previously [15]. Briefly, after exposure, the top and bottom surface of cells were covered with complete media. After 2.5 h the media in the top of the permeable support (contains detached/dead cells) was transferred to a 1.5-mL tube and centrifuged. A portion of the supernatant was transferred to a clean 1.5-mL tube and stored at − 80 °C for ELISA assay. To detach adherent/live cells from the permeable support, 0.25% trypsin (Corning) was added to the top and bottom of each well. After 10 min, complete media was added to the top of each permeable support and this media was mixed with the remaining supernatant and pellet. The media was then mixed with trypan blue dye (Corning), pipetted into a hemocytometer (Invitrogen) and measured in triplicate using a Countess cell counter (Invitrogen).

### Elisa

Six cytokines (IL-1β, IL-6, IL-10, CXCL1, CXCL2, and CXCL10) were measured as markers of cell inflammatory response using commercially available ELISA kits (CXCL2 Abcam, all others R&D Systems). For all assays, the manufacturer’s protocols were followed. Cytokine concentrations were adjusted for the number of live cells observed in the corresponding trypan blue assay.

### Statistical analysis

Statistical analysis was performed using Prism version 7.05 (GraphPad). Kruskal-Wallis non-parametric tests were performed for each study outcome to compare: 1) the mean rank of liquids vs. air controls 2) the mean rank of liquids vs. PG controls 3) the mean rank of NIC, CBD and NIC + CBD vs NIC, CBD and NIC + CBD. A Mann-Whitney t-test was performed to compare the statistical difference between NIC + CBD and NIC + CBD + Flavor. All experiments were performed in at least triplicate, with each outcome measured three times per experiment.

## Results

### GC/MS analysis of refill solutions

GCMS analysis showed that the primary cannabinoid in our products was CBD as listed on the packaging. Additionally, we found 2,3-butanediol, acetoin, acetone alcohol, benzaldehyde, and propylene glycol in the flavored commercial liquid, **Supplemental Table****1**.

### Effect of nicotine and CBD with and without flavor

#### PG only (PG, solvent control) exposure

Aerosols generated from various solutions (**PG, NIC, CBD, NIC + CBD and NIC + CBD + Flavor**) differed significantly in their toxicity on bronchial epithelial cells (Fig. 1). Metabolic activity decreased significantly compared to the air controls when cells were exposed to aerosols containing **PG** (*p* = 0.0101, Fig. 1a). When examining cytokine levels released after exposure to **PG** aerosols, we found a significant increase in IL-10 (*p* = 0.0081, Fig. 1e) compared to the air control.

#### PG + nicotine (NIC) exposure

Metabolic activity decreased significantly compared to the air controls when cells were exposed to aerosols containing **NIC** (*p* = 0.0009, Fig. 1a). When examining cytokine levels released after exposure to **NIC** aerosols, we found a significant increase in IL-1β (*p* = 0.0016, Fig. 1c) and IL-10 (*p* = 0.0005, Fig. 1e) compared to the air control.

#### PG + CBD (CBD) exposure

After exposure to aerosols containing **CBD**, metabolic activity and cell viability were significantly decreased compared to the air control (both assays *p* < 0.0001, Fig. 1a, b), as well as to the **PG** control *(p* < 0.0001 and *p* = 0.0088 respectively, Fig. 1a, b**)**. Aerosol containing **NIC** were found to be significantly different from aerosol containing **CBD** in both assays *(p* = 0.0007 and *p* = 0.0159 respectively, Fig. 1a, b). Additionally, exposure to **CBD** aerosol resulted in a significant increase in IL-1β, IL-10, CXCL1 and CXCL2 release compared to the air control *(p* < 0.0109, Fig. 1c-g), as well as compared to the **PG** control for CXCL1 (*p* = 0.0022, Fig. 1f). Finally, exposure to aerosol containing **NIC** resulted in significantly decreased release of cytokine CXCL2 compared to **CBD** aerosol *(p* = 0.0243, Fig. 1g).

#### PG + nicotine+CBD (NIC + CBD) exposure

When examining the effects of exposure to aerosol containing both **NIC + CBD**, we found a significant decrease in metabolic activity and cell viability compared to the air control (both assays *p* < 0.0001, Fig. 1a, b). In addition, we found a significant decrease in cell viability compared to **PG** control (*p* = 0.0012, Fig. 1b). Additionally, aerosol with **NIC + CBD** negatively affected cell viability compared to **NIC** condition (*p* = 0.0021, Fig. 1b). Metabolic activity of cells exposed to aerosol with **CBD** was also found to be significantly decreased compared to **NIC + CBD** condition (*p* = 0.0201, Fig. 1a). When examining the effect of exposure to **NIC + CBD** on inflammation, we found that IL-1β, IL-10, CXCL1, CXCL2 and CXCL10 were significantly increased compared to the air control (*p* < 0.0014, Fig. 1c-h). Similar differences were observed for **PG** control (*p* < 0.0103, Fig. 1c-h). Finally, exposure to aerosol containing **NIC + CBD** resulted in significant increase of cytokine release compared to **NIC** for IL-10, CXCL1, CXCL2 and CXCL10 (*p* < 0.0063, Fig. 1e-h).

#### PG + nicotine+CBD + flavor (NIC + CBD + Flavor) exposure

Cell viability and metabolic activity of H292 cells decreased significantly after exposure to aerosols from all liquids that contained **NIC + CBD + Flavor** compared to air (both assays *p* < 0.0001, Fig. 1a, b) and **PG** controls (*p* < 0.0001 and *p* = 0.0119, respectively, Fig. 1a, b). **NIC + CBD + Flavor** aerosol was found to have a significantly more deleterious effect on metabolic activity than unflavored **NIC + CBD** aerosol (*p* < 0.0001, Fig. 1a). Exposure to **NIC + CBD + Flavor** aerosol resulted in a significant increase in IL-1β, IL-6, IL-10, CXCL1, CXCL2 and CXCL10 levels compared to the air control (*p* < 0.0263, Fig. 1c-h). IL-1β, IL-6, CXCL1 and CXCL10 release was also significantly increased after exposure to **NIC + CBD + Flavor** aerosols compared to the solvent-only control (*p* < 0.0015, Fig. 1c-h). Exposure to **NIC + CBD + Flavor** aerosol resulted in increased production of IL-6, CXCL1 and CXCL10 (*p* < 0.0035, Fig. 1d-h) as well as a decrease in production of IL-10 (*p* = 0.0025, Fig. 1e*)* compared to unflavored **NIC + CBD** aerosol.

## Discussion

This pilot study used an in vitro model to examine potential respiratory effects of nicotine and CBD when co-administered together. Our data show that exposure to **NIC** containing liquids result in significant cytotoxic and inflammatory effects on the H292 bronchial epithelial cell line similarly to the effects observed after exposure to pure solvent (**PG**). These results are consistent with past in vitro studies that utilized a similar ALI exposure system [15, 18]. A novel finding is that exposure to **CBD** resulted in stronger cytotoxic and inflammatory effects compared to **NIC**.

Another novel finding is that co-exposure to nicotine and CBD (**NIC + CBD**) resulted in an additive cytotoxic effect on bronchial epithelial cells. This finding suggests that vapers who co-use nicotine and cannabinoid products may have increased risk of respiratory symptoms as compared to vapers who only use a single substance. Additionally, co-exposure of **NIC + CBD** aerosol resulted in an additive pro-inflammatory (IL-1β and chemokines, Fig. 1c, f-h) as well as additive anti-inflammatory (IL-10, Fig. 1e) response as compared to **NIC** or **CBD** aerosol. These results merit additional mechanistic studies to examine the effects of aerosolized CBD products on the inflammatory pathway. However, an important limitation of our study is that only one concentration of nicotine and CDB was utilized and we did not estimate dose-response effects. Further studies are needed to test these effects using varying nicotine and CBD concentrations to determine if these results are affected by drug concentration. Although we used a physiologically relevant ALI system, we did not measure any clinically relevant health outcome in ENDS users. Future in vivo studies are needed to determine if the effects of this study are applicable to human subjects.

Our study confirmed that addition of flavoring additives to liquid results in increased cytotoxic and inflammatory effects compared to unflavored products. These results reaffirm findings from previous studies that reported cytotoxic effects of various flavorings used in ENDS products [15, 19]. Additionally, the use of flavored e-cigarette liquids with **NIC + CBD** resulted in a significantly increased pro-inflammatory response as compared to the air and **PG** controls as well as compared to the **NIC + CBD** liquid without flavoring (IL-1β. IL-6 and Chemokines, Fig. 1c, d, f, h). This also resulted in a lowered anti-inflammatory response as compared to all other e-cigarette liquids in this study (IL-10, Fig. 1e). These results suggest that while **CBD** containing aerosol may produce an elevated pro-inflammatory response as compared to the **NIC** and **PG** solutions, that the largest factor that may result in e-cigarette use related inflammation is associated with co-exposure with flavoring agents. A limitation of this study is that only one flavor was utilized; thus, future studies are needed to test cytotoxic effects of products with different flavors. Another limitation of our study is that we did not examine whether decreased viability of cells had been a result of apoptosis or necrosis. Since we observed a significant increase in the pro-inflammatory cytokines/chemokines IL-1b, CXLC1, CXCL2 and CXCL10 and a significant increase in the anti-inflammatory cytokines (IL-10), we hypothesized that CBD aerosol was causing necrosis. However, it is also possible that CBD aerosol has caused apoptosis of these cells similar to the observations of Yu et al. 2016 [20]. Those alternative hypotheses should be tested in more comprehensive mechanistic studies in future.

## Conclusion

Our pilot in vitro study suggests a cumulative respiratory effect of inhaled nicotine and CBD. As co-use of nicotine and cannabis is increasing, studies are urgently needed to evaluate potential health consequences in users of both substances. This in vitro study suggests independent and additive toxic effects of vaporized nicotine and CBD further amplified by flavorings. With increased popularity of vaporized products, potential long-term respiratory effects need to be evaluated in those who vape nicotine and cannabinoids.

## Supplementary information

**Additional file 1: Supplemental Table 1.** Qualitative comparison of commercially purchased flavored e-cigarette liquids with and without CBD using gas chromatography. Liquid 1 (**Flavor**) contained propylene glycol and “Easy Rider” flavoring, while liquid 2 (**CBD + Flavor**) contained propylene glycol, 1.7 mg/ml CBD and “Easy Rider” flavoring. Qualitative detection of a compound is indicated with an X when identified in both National Institute of Standards and Technology (NIST) and Mass Spectra of Flavors and Fragrances of Natural and Synthetic Compounds (FFNSC) mass spectrometry libraries.

## Data Availability

The datasets used and/or analyzed during the current study are available from the corresponding author on reasonable request.

## Abbreviations

CBD: Cannabidiol
e-cigarette(s): Electronic cigarettes
ENDS: Electronic nicotine delivery systems
PG: Propylene glycol only
NIC: Nicotine
NIC + CBD: Nicotine with cannabidiol
NIC + CBD + Flavor: Nicotine, cannabidiol and flavoring
GC/MS: Gas chromatography/mass spectrometry
ALI: Air liquid interface
